# Metagenomic Analysis of Virioplankton from the Pelagic Zone of Lake Baikal

**DOI:** 10.3390/v11110991

**Published:** 2019-10-29

**Authors:** Sergey A. Potapov, Irina V. Tikhonova, Andrey Yu. Krasnopeev, Marsel R. Kabilov, Aleksey E. Tupikin, Nadezhda S. Chebunina, Natalia A. Zhuchenko, Olga I. Belykh

**Affiliations:** 1 Limnological Institute Siberian Branch of the Russian Academy of Sciences, Irkutsk, Ulan-Batorskaya 3, 664033, Russia; iren@lin.irk.ru (I.V.T.); andrewkrasnopeev@gmail.com (A.Y.K.); nchebun@lin.irk.ru (N.S.C.); zhna@lin.irk.ru (N.A.Z.); belykh@lin.irk.ru (O.I.B.); 2 Chemical Biology and Fundamental Medicine Siberian Branch of the Russian Academy of Sciences, Lavrentiev Avenue 8, Novosibirsk 630090, Russia; kabilov@niboch.nsc.ru (M.R.K.); alenare@niboch.nsc.ru (A.E.T.)

**Keywords:** virome, Lake Baikal, freshwater, viral ecology

## Abstract

This study describes two viral communities from the world’s oldest lake, Lake Baikal. For the analysis, we chose under-ice and late spring periods of the year as the most productive for Lake Baikal. These periods show the maximum seasonal biomass of phytoplankton and bacterioplankton, which are targets for viruses, including bacteriophages. At that time, the main group of viruses were tailed bacteriophages of the order Caudovirales that belong to the families *Myoviridae*, *Siphoviridae* and *Podoviridae*. Annotation of functional genes revealed that during the under-ice period, the “Phages, Prophages, Transposable Elements and Plasmids” (27.4%) category represented the bulk of the virome. In the late spring period, it comprised 9.6% of the virome. We assembled contigs by two methods: Separately assembled in each virome or cross-assembled. A comparative analysis of the Baikal viromes with other aquatic environments indicated a distribution pattern by soil, marine and freshwater groups. Viromes of lakes Baikal, Michigan, Erie and Ontario form the joint World’s Largest Lakes clade.

## 1. Introduction

Viral communities that inhabit the water column (virioplankton) represent the range of all viruses, including those that affect eukaryotes, as well as archaeal viruses and bacteriophages. Viruses can contain both RNA and DNA, and their numbers are comparable in aquatic environments [1]. Bacteriophages constitute a large part of viral communities [2] and are active participants in the microbial loop. They affect genetic diversity and control the number of heterotrophic bacteria and cyanobacteria, and this factor largely determines the content and structure of plankton [3].

The study of viral communities is difficult for several reasons. The primary reason is because only a small part of their hosts and viruses themselves can be cultivated. Moreover, because no one gene is common for all viral genomes, viral communities cannot be studied in the same way as bacteria and eukaryotes based on the analysis of 16S rRNA and 18S rRNA genes, respectively. Therefore, individual groups of viruses are studied by signature genes. For example, genes for capsid proteins, such as *g20* and *g23* [4,5,6], serve as targets for identification of T4-like myoviruses, the polA DNA polymerase gene [7] for podoviruses, the polB gene [8] for members of the family *Phycodnaviridae* and the RdRp gene [9,10] for RNA viruses.

Until recently, the focus of aquatic virologists was on the viromes of marine environments, whereas there were fewer studies of freshwater viromes. The first study of freshwater bodies investigated the viral communities in fish ponds [11]. Subsequently, studies characterised RNA viromes from Lake Needwood (Maryland, USA) [12] and the viral diversity from Lake Limnopolar (Antarctica) [13]. There were also metagenomic studies of viruses from two sites of an aquaculture facility [14], two lakes in France, Bourget and Pavin [15], four freshwater bodies located in the Sahara desert [16] and the Feitsui Reservoir in North Taiwan [17]. In 2013, metagenomic analysis of viral communities from East Lake (China) revealed high genetic diversity of viruses [18]. Recently, six arctic freshwater bodies in Spitsbergen (Svalbard, Norway) were studied and compared with an Antarctic lake. The authors revealed a taxonomic similarity in the investigated water bodies. However, the viromes differed at the fine-grain genetic level. These data indicate differences among dominant species of viruses. Single-stranded DNA (ssDNA) viruses predominated in Arctic viromes; most viruses remained unidentified [19]. The viromes of the Great Lakes, including Ontario and Erie, exhibited the dominance of bacteriophage sequences as well as a high content of plant and animal viruses; a comparative analysis indicated a similar composition of viruses in both lakes [20]. In the eutrophic Lake Matoaka (USA), sequences that belonged to tailed bacteriophages prevailed along with *Podoviridae* family members [21]. In the virome from Lake Michigan, like other similar viromes, most of the generated open reading frames (ORFs) were assigned to hypothetical proteins [22]. In Lake Lough Neagh (Northern Ireland), 85% of the virome did not have homologues in the extant sequence databases [23]. These data were consistent with most previous metagenomic analyses. Subsequent analysis of the annual dynamics in the content of the viral community indicated that 20% of the viruses were not detected during specific periods of the year [24].

To date, there have been no studies on sequencing viromes from the pelagic zone of Lake Baikal. Recent studies focused on the identification and genetic diversity of viruses of the *Tevenvirinae* subfamily (order Caudovirales, family *Myoviridae*) via the signature genes *g20* [25] and *g23* [26,27].

Lake Baikal is oligotrophic; it is the deepest lake in the world (1642 m) and has a large freshwater supply (2.36 × 10^4^ km^3^). Geological, geographical and hydrological characteristics of the lake reflect its uniqueness and high endemism of aquatic organisms [28]. Although some studies characterised viruses of Lake Baikal [25,26,29,30], their genetic diversity remains insufficiently investigated.

Spring is an important period in Lake Baikal. According to long-term data, this period corresponds to the highest algal biomass and rates of primary production [31,32,33]. The studied periods are regarded as early spring (under-ice period: February, March and April) and late biological spring (May and June) according to Kozhov [34]. The aim of this study was to determine the taxonomic composition of viral communities in the pelagic zone of Lake Baikal during the under-ice and late spring (open-water) periods, perform functional annotation of genes and comparative analysis with viromes previously obtained from other lakes and assemble contigs and compare them to the RefSeq and GenBank databases.

## 2. Materials and Methods

### 2.1. Sample Collection and Sequencing

For the concentration of the viral fraction and DNA extraction, ~25 L samples were taken on 22 March and 8 June 2018, 7 km and 3 km, respectively, from the Listvyanka settlement (southern basin of Lake Baikal). From each layer (0, 5, 10, 15, 20, 25 and 50 m), 3.5 L were sampled and mixed to obtain an integrated sample of the 0–50 m layer. Subsequently, the sample was filtered through polycarbonate filters with a pore size of 0.4 μm (Millipore, Burlington, MA, USA) to remove phyto- and zooplankton. The filtrate was concentrated using a VivaFlow 200 tangential flow ultrafiltration system (Sartorius, Göttingen, Germany) to the final volume of ~20 mL. It was passed through a nozzle with a pore size of 0.2 μm (Sartorius, Göttingen, Germany) to remove bacteria and concentrated using a Vivaspin Turbo 15 (50 kDa; Sartorius, Göttingen, Germany) to a volume of ~100 μL. The samples were processed for several hours; during filtration and concentration, the samples were refrigerated at 4 °C.

To obtain free virus particles, the sample was treated with DNase (1000 U/mL; Thermo Fisher Scientific, Waltham, MA, USA) at 37 °C for 30 min. DNase was inactivated by addition of 20 μL 50 mM ethylenediaminetetraacetic acid (EDTA) at 65 °C for 10 min [35]. The presence of bacterial DNA was examined by polymerase chain reaction (PCR) using universal bacterial primers 27L (5’-AGAGTTTGATCATGGCTCAG-3’) and 1542R (5’-AAGGAGGTGATCCAGCCS-3’) [36] to confirm the removal of external bacterial DNA. PCR showed the absence of bands.

The samples were processed with sodium dodecyl sulphate (SDS) and proteinase K. DNA was extracted using the standard phenol-chloroform method. The DNA concentration was measured using a Qubit 2.0 Fluorimeter (Invitrogen, Carlsbad, CA, USA), according to the manufacturer’s instructions (~50 ng of DNA was obtained). The extracted DNA was stored at −80 °C.

DNA was fragmented with a Covaris S2 (USA), and libraries were prepared using the NEBNext Ultra II DNA Library Prep Kit for Illumina (New England Biolabs, Ipswich, MA, USA). Whole genome amplification technology was not used. The obtained DNA libraries were sequenced on a MiSeq device (Illumina, San Diego, CA, USA) using the Kit v3 2 × 300 reagents (Illumina, San Diego, CA, USA) in the “Genomics Core Facility” (ICBFM SB RAS, Novosibirsk, Russia).

The samples were labelled as follows: 7 km from the Listvyanka settlement (March)–BVP1, and 3 km from the Listvyanka settlement (June)–BVP2; BVP1_2 indicated the cross-assembled sample.

### 2.2. Water Chemistry Analyses

The concentrations of total phosphorus, total nitrogen, total organic carbon, nitrate, nitrite and chlorophyll *a* were determined as previously described [33]. The concentration of oxygen was measured using the SBE 25 Sealogger CTD (Sea-Bird Electronics, Bellevue, Washington DC, USA).

### 2.3. Microbial Enumeration

Bacteria were quantified by epifluorescence microscopy using the fluorochrome 4’,6’-diamidino-2-phenylindole dihydrochloride (DAPI) [37]. Picoplanktonic cyanobacteria were quantified by phycobilin autofluorescence. The number of bacteria, cyanobacteria and virus particles was estimated by filtration using polycarbonate filters with a pore size of 0.2 μm (Sartorius, Göttingen, Germany). SYBR Green I fluorochrome [35] and 0.02 µm filters (Whatman, Maidstone, England) were used to detect virus particles. Bacteria and virus particles were counted on an Axio Imager M1 fluorescence microscope (Zeiss, Oberkochen, Germany) equipped with an HBO 100W mercury lamp and an AxioCam camera (Pixera Corp., Santa Clara, CA, USA) with a 100× magnification lens.

Samples (1 L volume) for qualitative analysis of phytoplankton were fixed with Lugol’s solution and then concentrated by sedimentation. Algae were counted twice in a 0.1 mL Nageotte chamber under a “Peraval” light microscope with 720× and 1200× magnification. Thin cells of *Synedra* *acus* subsp. *radians* (Kütz.) Skabitchevsky formed by the sexual process did not have silica valves, and thus 50 mL samples were filtered onto 1 μm pore-size “Millipore” polycarbonate filters and stained by DAPI for visualisation of nuclei and chloroplasts. Subsequently, the cells were counted directly on the filter using an Axio Imager M1 microscope with 200× magnification.

### 2.4. Bioinformatics Analysis

The virome was analysed with the online pipeline Meta Genome Rapid Annotation using Subsystem (MG-RAST); raw data were uploaded [38].

The assembly and analysis of contigs included the following stages. Quality control was performed using the Fast QC program (http://www.bioinformatics.babraham.ac.uk/projects/fastqc/). Then, the obtained data were processed with Trimmomatic v. 0.36 [39], using the parameter SLIDINGWINDOW:4:20. Sequences shorter than 50 nucleotides were excluded from the analysis; adapters were removed. The SPAdes 3.13.0 metagenomics assembler, metaSPAdes, with default parameters was used for the de novo assembly. All calculations were performed on the HPC-cluster “Akademik V.M. Matrosov” (“Irkutsk Supercomputer Centre of SB RAS, http://hpc.icc.ru”).

Contigs were assembled with SPAdes using the VirSorter (v. 1.0.3) tool. The contigs that belonged to viruses were sorted out (Virome database). Contigs that were shorter than 5 kilobase pairs (Kbp) were removed before processing. The length of contigs was chosen as the most optimal ratio of recall value to precision value. This value, according to the simulation, is also the minimum necessary for the correct identification of viral sequences [40]. Then, the closest homologues were determined using blastn (DB RefSeq 2019 and GenBank 2019), with an e-value parameter of 10^−^^3^. To predict genes, the MetaGeneMark tool [41] was used; subsequently, a search in the NR NCBI (2019) database was performed manually with blastp. The contig and genome architectures were drawn using EasyFig [42]. To check the coverage of the Baikal contigs with reads, the programmes BWA (v. 0.7.17; MEM algorithm, Li H., [43]) and SAMtools (v. 1.9) [44] were used. The result was visualised in the IGV genome browser (v. 2.4.14) [45].

A comparative analysis of viromes was performed using the online web server http://www.metagenassist.ca. The dendrogram was obtained from the comparison of the Baikal viromes with 23 viromes from different sources (bay, ocean [46], river [47], lakes [13,20,21,22,23], soil [48], hydrothermal fluid [49] and an aquaculture facility [11]; Supplementary Information Table S1). For input data, the operational taxonomic unit (OTU) tables with the assigned taxonomy were used. The parameters were as follows: Column-wise normalisation–Log (generalised log_2_ transformation), Analyse by taxonomy–genus, Distance Measure–Pearson, Clustering Algorithm–Average.

The original unprocessed reads were uploaded to the MG-RAST server (BVP1 ID mgm4814173.3, BVP2 ID mgm4816981.3) and NCBI (SRA project PRJNA547700).

## 3. Results

### 3.1. Environmental Characteristics

Table 1 shows temperature, Secchi disk transparency, concentrations of total phosphorus, organic carbonate and nitrogen and the content of oxygen, nutrients and chlorophyll *a* in the pelagic zone of Lake Baikal. Based on these data, the trophic state of Lake Baikal was assigned as oligotrophic with mesotrophic characteristics, according to the Vollenweider & Kerekes classification [50]. The diatom *Fragilaria radians* (Kützing) D.M. Williams and Round, also known as *S. acus* subsp. *radians* (Kützing) Skabitchevsky (Algae Base taxonomy [51]), dominated phytoplankton in March and June. The number of virus particles under the ice was slightly higher than during the open-water period. On the contrary, the total number of bacteria decreased in June compared to March (Table 1).

### 3.2. Overview of the Lake Baikal Virome

The obtained total “raw data” were as follows: BVP1, 3223426, and BVP2, 4,136,035 reads (2 × 300) of 301 nucleotides in length; GC-content was 43% and 44%, respectively (Table 2).

### 3.3. Taxonomic Composition

**Figure 1:**
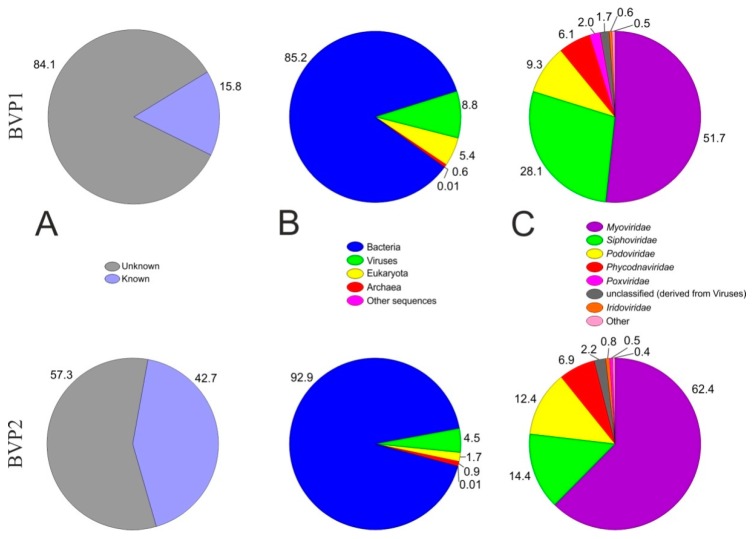
Content and structure of two viromes from Lake Baikal according to the RefSeq (MG-RAST) database (*e*-value 10^−5^). (**A**) The percentage of “known” virome sequences compared to the RefSeq database. (**B**) Breakdown of the “known” sequences into viruses, bacteria, archaea or eukarya using similarity results against RefSeq. (**C**) Taxonomic composition at the viral family level. The “Other” category pools families that represented less than 0.5% of the full virome sequences.

The bulk of sequences (84.1% for BVP1 and 57.3% for BVP2) did not show any similarity with sequences from the databases (Figure 1). This fact has been stated in all previously obtained viromes. We assigned 8.8% (BVP1) and 4.5% (BVP2) of all annotated sequences to the Viruses domain. 

The identified viruses mainly belonged to tailed bacteriophages of the order Caudovirales, which includes the families *Myoviridae*, *Siphoviridae* and *Podoviridae*. Among them, the *Myoviridae* phages predominated; they represented 51.7% (BVP1) and 62.4% (BVP2). The share of *Siphoviridae* was 28.1% (BVP1) and 14.4% (BVP2), and the contribution of *Podoviridae* was 9.3% (BVP1) and 12.4% (BVP2).

Overall, we identified 18 families of viruses that affect bacteria, algae, birds, fish, insects, humans, etc. *Myoviridae*, *Siphoviridae*, *Podoviridae*, *Phycodnaviridae* and *Poxviridae* comprised 97% of all identified families (Table 3).

The share of single-stranded viruses was 0.02% for BVP1 and 0.003% for BVP2.

### 3.4. Analysis of Sequences at the Genus Level

At the genus level, most annotated sequences belonged to T4-like viruses of the *Myoviridae* family. T4-like viruses are lytic bacteriophages; hence, this fact indicates a greater role of lytic phages in the plankton of Lake Baikal. 

At the species level, in March (BVP1), *Prochlorococcus* phage P-SSM2 (3374) had the highest number of hits in the *Myoviridae* family, followed by *Flavobacterium* phage 11b (1719), part of the *Siphoviridae* family, and *Synechococcus* phage Syn5 (227), a member of the *Podoviridae* family. In June (BVP2), there were *Prochlorococcus* phage P-SSM2 (9716), *Flavobacterium* phage 11b (701) and *Roseobacter* phage SIO1 (655), part of the *Podoviridae* family.

### 3.5. Functional Analysis

MG-RAST uses several databases for the functional annotation of reads, including four databases that allow for hierarchical functional annotation: Kyoto Encyclopaedia of Genes and Genomes (KEGG) Orthology (KO), Clusters of Orthologous Groups (COG), eggNOG and SEED Subsystems. We applied a functional classification based on SEED Subsystems. The database searches against SEED in the MG-RAST subsystem resulted in 69,486 (BVP1) and 363,448 (BVP2) hits. 

**Figure 2:**
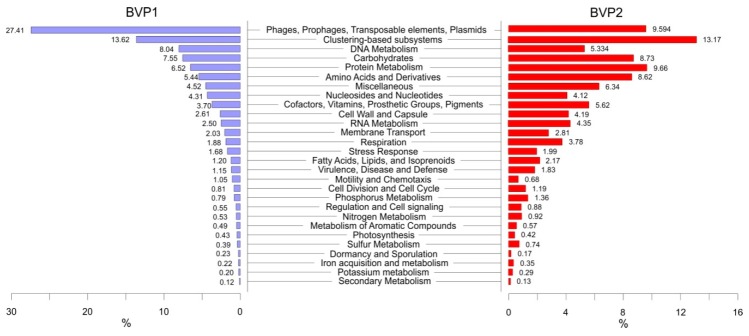
Functional annotations of the BVP1 and BVP2 viromes by SEED Subsystems (MG-RAST).

In the BVP1 sample, we identified 27.41% (19,044) of the classified reads as a part of the functional category “Phages, Prophages, Transposable elements, Plasmids” (Figure 2). These genes are associated with phage replication and packaging of virus particles (e.g., terminase, integrase, helicase and primase). “Phages, prophages” were the largest part of this group (97% of all classified reads in this group), whereas 1.3% of reads belonged to Gene Transfer Agents (GTA). In the functional category “Phages, Prophages, Transposable elements, Plasmids”, we assigned a small number of reads to the functional categories “Pathogenicity islands” (1.57%) and “Transposable elements and integrons” (0.16%). Notably, the subgroup with the most reads in the “Phages, prophages” category was “r1t-like streptococcal phages” (54.1%).

In the BVP2 sample, MG-RAST annotated 28 functional categories; each was subdivided into distinct subsystems. Some reads (9.6%, 34871) belonged to the functional category “Phages, Prophages, Transposable elements, Plasmids”. The largest part, 13.2% (47855), was the clustering-based subsystems category (e.g., biosynthesis of galactoglycans and related lipopolysaccharides; catabolism of an unclassified compound, etc. and other clusters identified as unclassified). The NULL subcategory included 29,684 sequences with the prevalence of Ribonucleotide reduction (3428) and Phosphate metabolism (3211) at level 3. The subgroup with the most reads in the “Phages, Prophages” category was “r1t-like streptococcal phages” (58.6%), similar to BVP1; phage protein and phage terminase annotations were most commonly identified.

### 3.6. Contig Analysis

The total number of obtained contigs was 255,462 for BVP1, 388,735 for BVP2 – 388,735 and 544,501 for BVP1_2 (cross-assembled). Further analysis included only contigs with a length of 5 Kb or more (Table 4).

After VirSorter processing, the number of contigs was 376 in BVP1, 776 in BVP2 and 1136 in cross-assembled BVP1_2. We annotated these contigs with blastn according to the Refseq 2019 and GenBank 2019 databases; the *e*-value was 10^−^^3^ (Table 5). At the first step, contigs were annotated using the Refseq database. Contigs that were not assigned to the closest relative at the first step were then annotated using the GenBank database. The remaining contigs were not assigned to the closest relative because they had very low similarity. 

In the BVP1 sample, we annotated 242 contigs (156 RefSeq and 86 GenBank) of 376; 34 contigs had the closest cyanophage relative (*Synechococcus* or *Prochlorococcus*; 66.0–84.8% similarity); 13 contigs were similar to Yellowstone Lake virophage (66.9–86.7% similarity); 14 contigs belonged to *Cellulophaga* phage (70.2–88.4% similarity) and four contigs were Pelagibacter phages (73.0–86.4% similarity). Among the uncultivated viruses, the majority of hits belonged to Dishui Lake virophage 1, 11 contigs (67.6–79.7% similarity).

In the BVP2 sample, we annotated 527 contigs (377 RefSeq and 150 GenBank) of 776, among which 111 contigs had the highest similarity with cyanophages (66.4–87.1% similarity). Additionally, 20 contigs belonged to the Yellowstone Lake virophages (66.5–86.7% similarity), 18 contigs to the Pelagibacter phages (64.7–81.4% similarity) and 15 contigs to the *Cellulophaga* phage (72.7–88.4% similarity). Finally, 51 contig belonged to uncultivated Mediterranean phage (65.0–86.0% similarity).

In the cross-assembled BVP1_2 sample, we annotated 772 contigs (538 RefSeq and 234 GenBank) of 1136. A total of 151 contigs had the highest similarity with cyanophages (63.2–92.7% similarity), 36 contigs belonged to the Yellowstone Lake virophages (66.5–86.7% similarity), 20 contigs were part of Pelagibacter phages (65.4–90.4% similarity) and 39 contigs belonged to *Cellulophaga* phage (67.9–94.9% similarity). Finally, 81 contig belonged to uncultivated Mediterranean phage (63.2–84.7% similarity).

The number of annotated contigs with a length greater than 25 Kbp was 16 in BVP1, 31 in BVP2 and 54 in cross-assembled BVP1_2.

The BWA program was used to map back to the raw data. For BVP1, 14% of reads were mapped to VirSorter contigs, and 12.8% of reads for BVP2.

Table 5 shows the first 10 contigs for each assembly with a minimum e-value. Other annotated contigs are shown in Supplementary Table S2.

**Figure 3:**
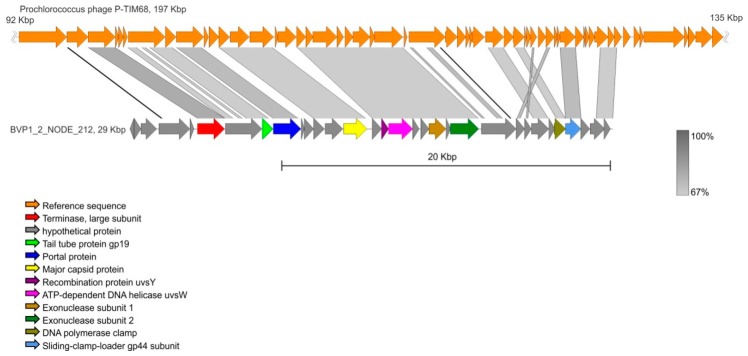
Comparison of BVP1_2_ NODE_212 contig and *Prochlorococcus* phage P-TIM68.

Figure 3 shows the genetic map of the BVP1_2_NODE_212 contig. Based on the analysis of the terminase large subunit, we classified this phage as closely related to the cultured marine *Synechococcus* phage Bellamy (MF351863) and uncultured Mediterranean phage (KT997817). According to the results of the MetaGeneMark analysis, the number of predicted ORFs was 32. We visualised the contig coverage with reads in the IGV genome browser (Supplementary Information Figure S1). Visualisation indicated no gaps and uniform coverage.

### 3.7. Comparative Analysis of Viromes

**Figure 4:**
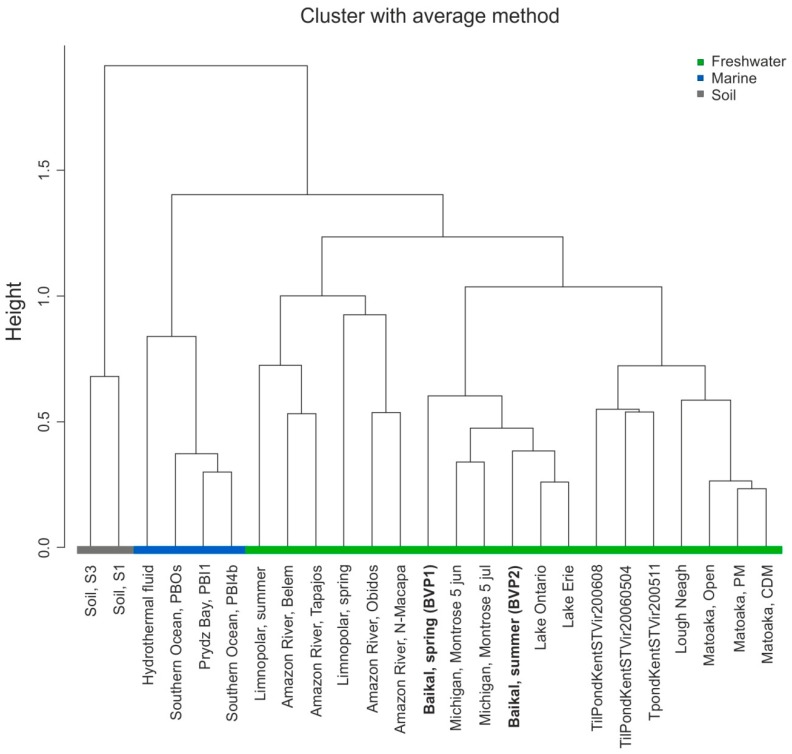
Agglomerative hierarchical clustering tree for the comparative analysis of viromes.

A dendrogram constructed with the agglomerative hierarchical clustering demonstrated a clear separation of viromes into groups: Soil, marine and freshwater (Figure 4). Viromes from Lake Baikal (BVP1 and BVP2) form a cluster with viromes of the world’s largest lakes (Michigan, Ontario and Erie). BVP1 has a separate branch in this cluster. BVP2 is located closer to viromes from Lakes Ontario and Erie. Viromes from Lakes Matoaka and Lough Neagh, as well as fish ponds, form a eutrophic cluster. Table 6 shows the main characteristics of the lakes.

## 4. Discussion

Using high-throughput sequencing, we obtained the first data on the content and structure of viral communities from the pelagic zone of Lake Baikal, the world’s oldest and largest lake in terms of area and water volume.

The Lake Baikal pelagic zone is an oligotrophic water body, as stated previously [52] and confirmed by our data on the concentration of total phosphorus, nitrogen, chlorophyll *a* content and water transparency. In May and June, there is a steady stratification of the water column that corresponded to the spring period (inverse thermal stratification). At Lake Baikal, early June refers to the period of biological spring. Water chemistry characteristics in March and June 2018 were similar in the interannual aspect compared to the previous years of observations; no changes were found. Notably, under the ice, the development level of the diatom *F. radians*, which dominated the plankton of the lake, was slightly higher compared to June, as was the concentration of chlorophyll *a*. In general, the concentration of chlorophyll *a* corresponded to the characteristics we previously observed in March and June 2016 at the same station [33]. The total number of virus particles and bacteria was similar to the same periods of other years [33,53]. 

Although the viral sequences in the two viromes showed significant and distinct diversity, they were similar in taxonomic composition; bacteriophages of the order Caudovirales (89.3%) dominated. In other freshwater bodies, viruses from the order Caudovirales also predominated among the virus fraction. Lake Limnopolar, lakes of the Svalbard archipelago and Lakes Bourget and Pavin are the exception. Bacteriophages can be easily isolated from natural samples and sequenced, and thus their sequences predominate in databases. This fact explains their prevalence in viromes [22], and our data support this statement.

In virioplankton of Lake Baikal, the bulk of the order Caudovirales comprised phages that belong to the *Myoviridae* family. The members of this family were also the most numerous in Lakes Erie and Ontario [20], East Lake (Aug 2009, Dec 2009, and June 2010) [18], Lake Michigan [22], in tropical water bodies of Singapore [54] and freshwater bodies of the Sahara desert [16]. 

Sequences of the Baikal viromes annotated from databases were mostly bacterial (85.2% for BVP1 and 92.9% for BVP2). The bacterial part is probably overvalued due to the erroneous classification of metavirome sequences of prophages because, according to databases, they are of bacterial origin [55]. 

Of special interest is a small number of viruses with ssDNA in Lake Baikal; their content was 0.02% (BVP1) and 0.003% (BVP2). The same low content was noted in Lake Lough Neagh (0.5%). On the contrary, the share of ssDNA viruses in Lakes Pavin and Bourget was 80% and 85%, respectively [15]. In the spring sample, ssDNA viruses dominated viromes of Arctic lakes (74%); in the summer sample, they were 9% [19]. This distinction is due to the difference in the preparation of metagenomic libraries for sequencing [56,57]. In most studies, sampling for virome sequencing was performed from the surface water layers (0–5 m). In some cases, the shallowness of the water body limited the sampling depth.

Notably, sequences of the viral fraction were mostly dissimilar to the sequences from databases [13,20]. This so-called “viral dark matter” [58] represents content and structure that remain unknown in all studied samples [18,20,21,23].

After assembling the reads, there were no whole genome sequences that exhibited significant similarity to the reference sequences. Long contigs (over 25 Kbp) represented 5% (BVP1), 5% (BVP2) and 5.6% (BVP1_2) of the sequences that belonged to viruses after VirSorter processing. The identified pool of the Baikal contigs probably represents endemic Baikal viruses, or it is associated with a small number of reference genomes from freshwater bodies in the database. In general, the cross-assembled method yields longer contigs.

The BVP1_2_NODE_212 contig was the most similar to the reference sequences and had a great number of known genes. According to the RefSeq database, *Prochlorococcus* phage P-TIM68 was the closest relative of this contig. Although *Prochlorococcus* species are marine cyanobacteria, previous studies determined that *Prochlorococcus* phages may contain several genes that are similar to other T4-type viruses. They are common for all cyanophages, and in our case the same picture is likely [59]. For example, in Lake Michigan many annotated viral proteins belong to *Prochlorococcus* phage P-SSM2 [22]. 

Comparative analysis of the Baikal viromes in functional categories demonstrated that the “Phages, Prophages, Transposable elements, Plasmids” category prevailed during the under-ice period. The predominance of this category likely indicates the active replication (reproduction) of viruses under the ice. There are more bacteria during this period. In the late spring, the clustering-based subsystems category contained the most sequences, together with the NULL subcategory, which represented the bulk of sequences from this category. The NULL subcategory may contain some wrong assignments, and this possibility may indicate either the uniqueness or lack of sequences with known functions in the SEED database.

Agglomerative hierarchical clustering of viromes clearly separated viromes into freshwater, soil and marine groups. Studies by other authors confirm such clustering [15,21].

On the dendrogram, the Baikal viromes are most closely located near viromes from the world’s other largest lakes in terms of area, such as Ontario, Erie and Michigan, and form the joint World’s Largest Lakes (WLL) clade. Lakes Michigan, Erie and Ontario are a part of the Great Lakes system in North America; they are located in temperate climate zone, like Lake Baikal. Lake Michigan was previously mesotrophic; currently, its trophic state is defined as oligotrophic [60]. Lake Erie is mesotrophic, and Lake Ontario is oligo-mesotrophic. Obviously, combining these viromes into the WLL cluster reflects several characteristics, including morphometry, geographical location and trophic state, all of which determine the content and structure of viral communities. Centric diatoms, primarily, members of the genera *Aulacoseira* and *Stephanodiscus*, are the drivers of the spring planktonic communities in the Great Lakes. In Lake Baikal, the complex of endemic diatoms, mainly of *Aulacoseira baicalensis* and *Stephanodiscus meyeri*, dominated under the ice. However, since 2007 Baikal phytoplankton have shown a change in the diatom communities: A smaller and weakly silicified diatom *F. radians* (same as *S. acus*) has dominated the spring plankton of the lake [33].

Similar to Lake Baikal, most viruses in the viromes of Lake Michigan belong to the order Caudovirales, inside which the families *Myoviridae*, *Siphoviridae* and *Podoviridae* dominate (27%, 23% and 9%, respectively, of the total number of known virus sequences) [22]. In viromes of Lakes Erie and Ontario, the double-stranded DNA (dsDNA) phages of the order Caudovirales were also the most numerous; the *Myoviridae* family predominated (79.7%). *Podoviridae* (7.9%) and *Siphoviridae* (4.5%) were the second and third predominant families [20]. Concomitantly, viruses that affect algae (the *Phycodnaviridae* family; 4.3%) and insects (and animals; the *Iridoviridae* family; 2.6%) were the most representative. Notably, viromes studied in Lakes Ontario, Erie and Michigan were sampled from the surface in the coastal area. In our case, we obtained viromes from the pelagic layer of 0–50 m. Nevertheless, they formed a joint cluster. As mentioned above, a separate branch represents BVP1 (under-ice period) in this cluster. The seasonal taxonomic shift can explain the observed nature of the separation of the Baikal viromes and their position on the dendrogram because viromes from Lakes Michigan, Ontario, Erie, and BVP2 were sampled in the summer, namely June and July.

Two clusters comprised the neighbouring eutrophic clade: Viromes from fish ponds and viromes from eutrophic shallow lakes Lough Neagh (Ireland) and Matoaka (USA). 

River viromes, which group with viromes from Lake Limnopolar, represent a separate branch of the freshwater clade. This clustering is likely due to the similar content of the dominant virus families. In Lake Limnopolar, ssDNA viruses of the families *Circoviridae*, *Nanoviridae* and *Microviridae* prevail in the spring period, whereas in the summer period, *Phycodnaviridae* and bacteriophages of the order Caudovirales prevail. The families *Microviridae* and *Myoviridae* dominate in samples from the Amazon River. Viruses from the families *Circoviridae*, *Podoviridae*, *Phycodnaviridae* and *Siphoviridae* were also numerous.

After a comparative analysis of the results, we concluded that methodological specifics of sample preparation for high-throughput sequencing, read depth and bioinformatics data processing are crucial. Standardisation of virome processing requires platforms with an installed pipeline, such as MG-RAST, to avoid problems with different settings of processing programmes, sequence annotation and evaluation of alpha and beta diversity. The presence of bacterial DNA, as well as ultramicrobacteria (less than 2 μm in size) that can pass through a 0.2 μm filter, significantly complicates the analysis of viromes. In this study, we used prefiltration through filters with a pore size of 0.2 μm. The separation of virus particles in a gradient of caesium chloride [35] or chemical flocculation [61] may be possible alternatives. Notably, there is a lack of data on the whole genomes of viruses. The isolation and cultivation of virus strains followed by the characterisation of their genomes are an important component for future studies.

## 5. Conclusions

For the first time, we characterised the content of viruses in the pelagic zone of Lake Baikal, the world’s oldest and largest lake. The data showed the presence of a significant number of bacteriophage taxa compared to eukaryotic and archaeal viruses. Members of the order Caudovirales dominated the bacteriophages, with the prevalence of the *Myoviridae* family; *Siphoviridae* and *Podoviridae* were the second and third dominant families, respectively. We assembled the contig with a length of 29,000 bp, which belongs to cyanophage. A comparative analysis of viromes indicated their separation into marine, freshwater and soil clades and allowed us to assign the WLL cluster, which includes viral communities of Lake Baikal and the Great Lakes of North America. 

## Figures and Tables

**Table 1:** Characteristics during the sampling periods (mean values for 0–50 m layer, except for transparency).

Water Property	BVP1	BVP2
Water temperature (°C)	0.4–1.3 (0.75*)	2.7–2.8 (2.76)
pH	7.92–7.98 (7.95)	7.75–7.82 (7.79)
N_total_ (mg/L)	0.17–0.31 (0.23)	0.20–0.34 (0.29)
P_total_ (µg/L)	11–15 (13)	10–12 (11)
TOC (mg/L)	0.7–1.3 (1.07)	1.7–1.9 (1.8)
NO_2_^−^ (mg/L)	0.001 (the entire layer)	0.001–0.003 (0.002)
NO_3_^−^ (mg/L)	0.34–0.45 (0.39)	0.37–0.40 (0.39)
O_2_ (mg/L)	13.5–14.8 (14.3)	12.6–12.8 (12.7)
PO_4_^3−^ (µg/L)	24–40 (30)	22–26 (24)
Chl*a* (µg/L)	0.65–3.42 (1.83)	1.31–1.59 (1.40)
Viruses (VLPs mL^−1^)	2 (±1) × 10^6^	1.9 (±0.8) ×10^6^
Bacteria (cell mL^−1^)	1.5 (±0.7) × 10^6^	0.19 (±0.3) × 10^6^
Transparency, m	11	16

* arithmetical mean.

**Table 2:** Sequencing summary statistics for each virome (the number of reads is indicated).

Sample	Raw Data	Uploaded to MG-RAST	Annotated, RefSeq	Sequences Containing Ribosomal RNA Genes
BVP1	3223426	1474135	233310	929
BVP2	4136035	1956295	835350	2675

**Table 3:** Virus families in the BVP1 and BVP2 viromes.

Virus Family	Primary Host	Relative Abundance (% of Viral Sequences)	
		BVP1	BVP2
*Myoviridae*	Bacteria	51.7	62.4
*Siphoviridae*	Bacteria	28.1	14.4
*Podoviridae*	Bacteria	9.3	12.4
*Phycodnaviridae*	Algae	6.1	6.9
*Poxviridae*	Birds, mammals, humans	2	0.5
Unclassified viruses	-	1.7	2.2
*Iridoviridae*	Insects, amphibians, fish, invertebrates	0.5	0.8
Unclassified (Caudovirales)	Bacteria	0.2	0.2
*Baculoviridae*	Insects	0.2	0.1
*Marseilleviridae*	Amoeba	0.08	0.09
*Microviridae*	Bacteria	0.02	0.003
*Nimaviridae*	Crustaceans	0.02	0.01
*Herpesviridae*	Animals, including humans	0.02	0.04
*Polydnaviridae*	Insects	0.02	-
*Ascoviridae*	Invertebrates	0.01	0.003
*Asfarviridae*	Insects, pigs	0.01	0.01
*Lipothrixviridae*	Archaea	0.01	0.003
*Circoviridae*	Birds, mammals	0.005	-
*Parvoviridae*	Warm-blooded animals, humans	0.005	-
*Alloherpesviridae*	Fish, amphibians	0.005	0.008

**Table 4:** Data on the obtained contigs.

Sample	Number of Contigs Assembled	Max Length (bp)	Median	Number of Contigs ≥ 5 Kbp
BVP1	25,5462	127,498	326	1383
BVP2	388,735	1,129,755	425	3041
BVP1_2	544,501	1,129,000	367	4438

**Table 5:** Blast analysis of contigs identified in the Lake Baikal viromes.

Contig	Length (bp)	Number of Identified Open Reading Frames (ORFs)	Best BLAST Hit Affiliation	Accession Number	% Identity	Query Cover (%)
BVP1_NODE_544	9190	8	Yellowstone Lake virophage 7	NC_028257	75.92	34
BVP1_NODE_724	7752	9	Pelagibacter phage HTVC010P	NC_020481	73.05	88
BVP1_NODE_937	6565	8	*Synechococcus* phage S-SM2	NC_015279	70.20	89
BVP1_NODE_1041	6082	6	*Synechococcus* phage S-RIP2	NC_020838	71.18	69
BVP1_NODE_1110	5801	8	*Synechococcus* phage S-SM2	NC_015279	72.93	84
BVP1_NODE_667	8107	18	*Synechococcus* phage S-CBS4	NC_016766	67.78	65
BVP1_NODE_697	7967	12	Flavobacterium phage 11b	NC_006356	71.85	48
BVP1_NODE_1160	5626	12	*Staphylococcus* phage G1	NC_007066	99.77	99
BVP1_NODE_1162	5621	7	*Synechococcus* phage S-SM2	NC_015279	72.23	82
BVP1_NODE_1352	5081	9	*Staphylococcus* phage Sb-1	NC_023009	99.98	100
BVP2_NODE_1582	7385	10	*Synechococcus *phage S-SM2	NC_015279	74.52	90
BVP2_NODE_1722	7059	8	*Synechococcus* phage S-SM2	NC_015279	70.49	88
BVP2_NODE_1766	6972	10	*Synechococcus* phage S-CBS4	NC_016766	72.36	55
BVP2_NODE_2275	5991	4	*Prochlorococcus* phage P-SSM2	NC_006883	74.59	73
BVP2_NODE_2795	5268	7	*Synechococcus* phage S-SM2	NC_015279	71.99	74
BVP2_NODE_2816	5244	7	*Prochlorococcus* phage P-SSM2	NC_006883	73.91	81
BVP2_NODE_3036	5004	7	Pelagibacter phage HTVC010P	NC_020481	80.00	89
BVP2_NODE_344	17331	10	*Synechococcus* phage S-SM2	NC_015279	72.75	55
BVP2_NODE_721	11566	20	*Synechococcus* phage S-CAM9	NC_031922	67.18	40
BVP2_NODE_969	9692	9	*Synechococcus* phage S-SKS1	NC_020851	70.84	82
BVP1_2_NODE_831	13691	17	*Synechococcus* phage S-SM2	NC_015279	74.40	85
BVP1_2_NODE_1425	10187	9	*Synechococcus* phage S-SM2	NC_015279	70.55	78
BVP1_2_NODE_1820	8894	9	*Synechococcus* phage S-RIP2	NC_020838	71.18	55
BVP1_2_NODE_3506	5863	11	*Synechococcus *phage S-CBS4	NC_016766	67.83	75
BVP1_2_NODE_3812	5557	8	*Prochlorococcus* phage P-SSM2	NC_006883	73.86	83
BVP1_2_NODE_212	29427	32	*Prochlorococcus* phage P-TIM68	NC_028955	68.58	64
BVP1_2_NODE_496	18281	20	*Synechococcus* phage S-SKS1	NC_020851	65.93	27
BVP1_2_NODE_721	14952	5	*Synechococcus* phage S-SM2	NC_015279	72.46	58
BVP1_2_NODE_856	13532	12	*Synechococcus* phage S-SKS1	NC_020851	70.82	81
BVP1_2_NODE_996	12347	17	Pelagibacter phage HTVC010P	NC_020481	82.53	77

**Table 6:** Viromes from lakes used for agglomerative hierarchical clustering (includes the data source).

Name	Trophic Level	Average Depth (m)	Area (km^2^)	Country	Sampling Data
Lake Michigan	oligotrophic	279 281 (max)	58,030	USA	06.13 07.13
Lake Baikal	oligotrophic with mesotrophic characteristics	744.7 1681 (max)	31,722	Russia	This study
Lake Erie	mesotrophic	19 64 (max)	25,700	USA, Canada	07.13
Lake Ontario	oligo-mesotrophic	86 244 (max)	19,500	USA, Canada	06.13
Lough Neagh	eutrophic	9 31 (max)	392	Northern Ireland	04.14
Lake Matoaka	eutrophic	2.5 4.75 (max)	0.16	USA	03.13
Lake Limnopolar	ultra-oligotrophic	5.5 (max)	0.02	Antarctica	11.06 01.07

## Supplementary Materials

The following are available online at https://www.mdpi.com/1999-4915/11/11/991/s1, Supplementary Information, Table S1: Viromes used in building dendrogram; Table S2: Annotated contigs, Figure S1: Coverage contig BVP1_2 _212 by reads.
