# Supplementary material for: Metagenomic Analysis of Virioplankton from the Pelagic Zone of Lake Baikal
Source: Viruses. 2019 Oct 29;11(11):991. doi: 10.3390/v11110991 (PMC6893740; doi:10.3390/v11110991)
Supplement: Supplementary file 1 [file viruses-11-00991-s001.zip › Supplementary Table 1.docx]

Supplementary Table S1 Viromes used in building dendrogram

| MG-RAST and SRA ID | Sample | Source | Reference |
| --- | --- | --- | --- |
| mgm4684109.3 | S1 | Soil | [1] |
| mgm4689539.3 | S3 | Soil |  |
| mgm4448226.3 | 0.2-um-passable microorganisms | Hydrothermal fluid | [2] |
| mgm4747909.3 | PBOs | Prydz Bay | [3] |
| mgm4747904.3 | PBI1 |  |  |
| mgm4747910.3 | PBI4b |  |  |
| mgm4441558.3 | Summer | Lake Limnopolar | [4] |
| mgm4441778.3 | Spring |  |  |
| mgm4559916.3 | Belém | Amazon River | [5] |
| mgm4559927.3 | Tapajós |  |  |
| mgm4559919.3 | Óbidos |  |  |
| mgm4559917.3 | North Macapá |  |  |
| SRR1302010 | Montrose, 5 June 2013 | Lake Michigan | [6] |
| SRR1302020 | Montrose, 5 July 2013 |  |  |
| SRR2083227 | Lakeside-4_VD | Lake Ontario | [7] |
| SRR2083220 | Long Beach-4_VD | Lake Erie | [7] |
| mgm4440424.3 | TilPondKentSTVir200608 | Tilapia aquaculture facility | [8] |
| mgm4440412.3 | TilPondKentSTVir20060504 |  |  |
| mgm4440439.3 | TpondKentSTVir200511 |  |  |
| mgm4585272.3 | 4pW | Lough Neagh | [9] |
| mgm4523576.3 | Matoaka Open (MO) | Lake Matoaka | [10] |
| mgm4523575.3 | Pogonia Mouth (PM) |  |  |
| mgm4523574.3 | Crim Dell Mouth (CDM) |  |  |

1. Yu, D.; Han, L. Diversity and Distribution Characteristics of Viruses in Soils of a Marine-Terrestrial Ecotone in East China. **2018**, 375–386, doi:10.1007/s00248-017-1049-0.

2. Nakai, R.; Abe, T.; Takeyama, H.; Naganuma, T. Metagenomic Analysis of 0.2-μm-Passable Microorganisms in Deep-Sea Hydrothermal Fluid. *Mar. Biotechnol.* **2011**, *13*, 900–908, doi:10.1007/s10126-010-9351-6.

3. Mcminn, A.; Gong, Z.; Liang, Y.; Wang, M.; Jiang, Y.; Yang, Q.; Xia, J. Viral Diversity and Its Relationship With Environmental Factors at the Surface and Deep Sea of Prydz Bay , Antarctica. **2018**, *9*, 1–17, doi:10.3389/fmicb.2018.02981.

4. López-Bueno, A.; Tamames, J.; Velázquez, D.; Moya, A.; Quesada, A.; Alcamí, A. High diversity of the viral community from an Antarctic lake. *Science* **2009**, *326*, 858–861, doi:10.1126/science.1179287.

5. Silva, B.S. de O.; Coutinho, F.H.; Gregoracci, G.B.; Leomil, L.; de Oliveira, L.S.; Fróes, A.; Tschoeke, D.; Soares, A.C.; Cabral, A.S.; Ward, N.D.; et al. Virioplankton assemblage structure in the lower river and ocean continuum of the Amazon. *mSphere* **2017**, *2*, e00366-17, doi:10.1128/mSphere.00366-17.

6. Watkins, S.C.; Kuehnle, N.; Ruggeri, C.A.; Malki, K.; Bruder, K.; Elayyan, J.; Damisch, K.; Vahora, N.; O’Malley, P.; Ruggles-Sage, B.; et al. Assessment of a metaviromic dataset generated from nearshore Lake Michigan. *Mar. Freshw. Res.* **2016**, *67*, 1700, doi:10.1071/MF15172.

7. Mohiuddin, M.; Schellhorn, H.E. Spatial and temporal dynamics of virus occurrence in two freshwater lakes captured through metagenomic analysis. *Front. Microbiol.* **2015**, *6*doi:10.3389/fmicb.2015.00960.

8. Dinsdale, E. a; Edwards, R. a; Hall, D.; Angly, F.; Breitbart, M.; Brulc, J.M.; Furlan, M.; Desnues, C.; Haynes, M.; Li, L.; et al. Functional metagenomic profiling of nine biomes. *Nature* **2008**, *452*, 629–632, doi:10.1038/nature07346.

9. Skvortsov, T.; De Leeuwe, C.; Quinn, J.P.; McGrath, J.W.; Allen, C.C.R.; McElarney, Y.; Watson, C.; Arkhipova, K.; Lavigne, R.; Kulakov, L.A. Metagenomic characterisation of the viral community of lough neagh, the largest freshwater lake in Ireland. *PLoS One* **2016**, *11*, 1–19, doi:10.1371/journal.pone.0150361.

10. Green, J.C.; Rahman, F.; Saxton, M.A.; Williamson, K.E. Metagenomic assessment of viral diversity in lake matoaka, a temperate, eutrophic freshwater lake in southeastern Virginia, USA. *Aquat. Microb. Ecol.* **2015**, *75*, 117–128, doi:10.3354/ame01752.
