# Supplementary figures and images for: Metagenomic Analysis of Virioplankton from the Pelagic Zone of Lake Baikal
Source: Viruses. 2019 Oct 29;11(11):991. doi: 10.3390/v11110991 (PMC6893740; doi:10.3390/v11110991)

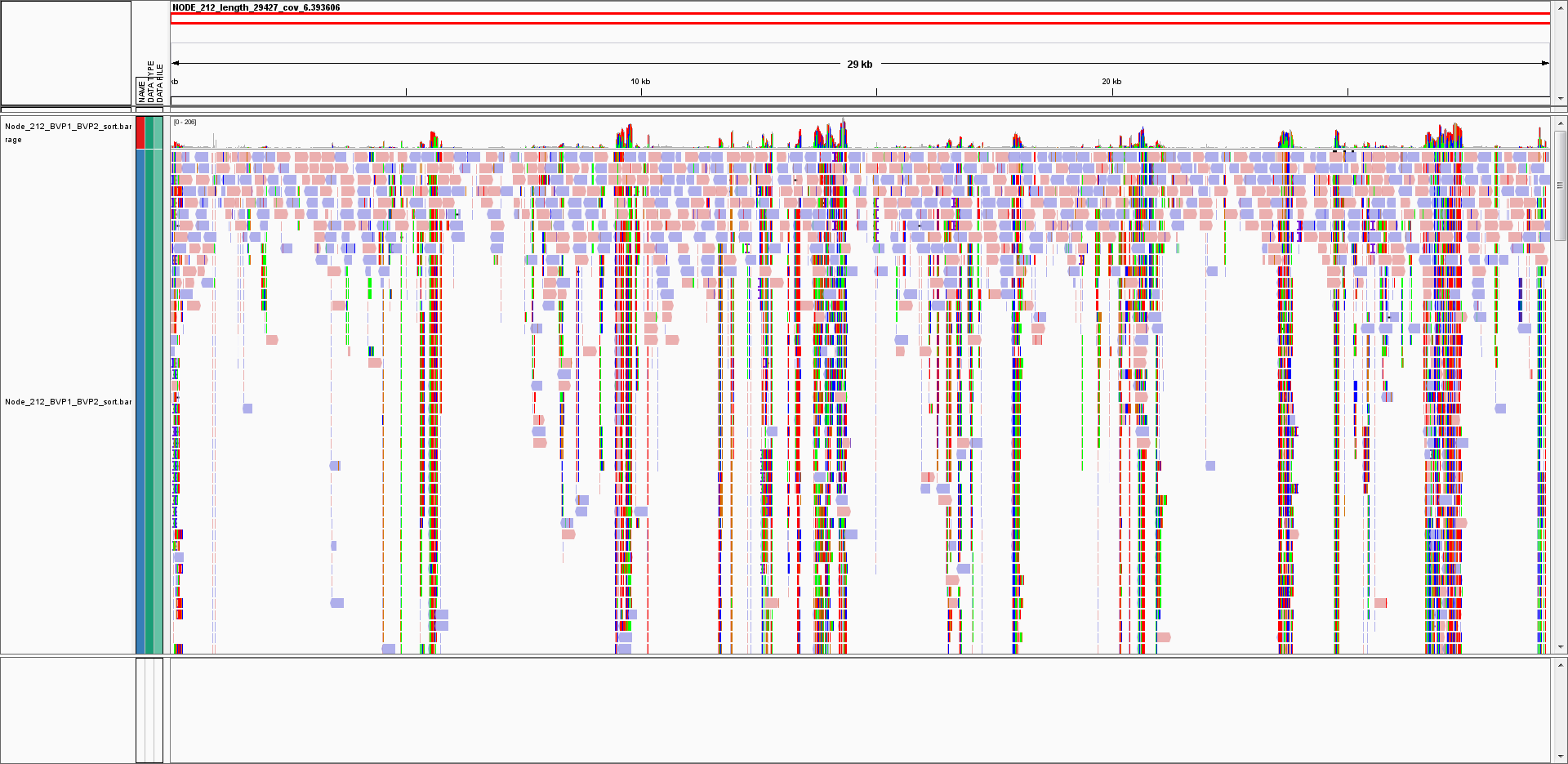

Supplement: Supplementary file 1 [file viruses-11-00991-s001.zip › Supplementary Fig. S1.png]
